# Identification of Biomarkers Associated with Diagnosis of Osteoarthritis Patients Based on Bioinformatics and Machine Learning

**DOI:** 10.1155/2022/5600190

**Published:** 2022-06-13

**Authors:** Yihao Liang, Fangzheng Lin, Yunfei Huang

**Affiliations:** ^1^Department of Orthopedics, The Second Clinical College of Guangzhou University of Chinese Medicine, Guangzhou, China; ^2^The Second Clinical College of Guangzhou University of Chinese Medicine, Guangzhou, China; ^3^Spine Surgery, The Honghui Hospital, Xi'an Jiaotong University, Xi'an, China

## Abstract

Osteoarthritis (OA) is thought to be the most prevalent chronic joint disease. The incidence of OA is rising because of the ageing population and the epidemic of obesity. This research was designed for the identification of novel diagnostic biomarkers for OA and analyzing the possible association between critical genes and infiltrated immune cells. 10 OA samples from patients with spinal OA and 10 normal samples were collected. GSE55235 and GSE55457 datasets including human OA and normal samples were downloaded from the GEO datasets. Differentially expressed genes (DEGs) were identified between 20 OA and 20 controls. SVM-RFE analysis and LASSO regression model were carried out to screen possible markers. The compositional patterns of the 22 types of immune cell fraction in OA were determined by the use of CIBERSORT. The expression level of the biomarkers in OA was examined by the use of RT-PCR. In this study, an overall 44 DEGs were identified: 18 genes were remarkably upregulated and 26 genes were distinctly downregulated. KEGG pathway analyses revealed that pathways were significantly enriched including IL-17 signal path, rheumatoid arthritis, TNF signal path, and lipid and atherosclerosis. Based on the results of machine learning, we identified APOLD1 and EPYC as critical diagnostic genes in OA, which were further confirmed using ROC assays. Immune cell infiltration analysis revealed that APOLD1 was correlated with mastocytes stimulated, NK cells resting, T cells CD4 memory resting, DCs stimulated, T cells gamma delta, macrophages M0, NK cells stimulated, and mastocytes resting. Moreover, we found that EPYC was correlated with mastocytes stimulated, NK cells resting, T cells CD4 memory resting, DCs stimulated, T cells gamma delta, macrophages M0, NK cells stimulated, and mastocytes resting. Overall, our findings might provide some novel clue for the exploration of novel markers for OA diagnosis. The critical genes and their associations with immune infiltration may offer new insight into understanding OA developments.

## 1. Introduction

Osteoarthritis (OA) is a commonly seen degeneration illness associated with age, corpulency, sex, bodyweight, and trauma [1]. It is featured by synovial lesion, osteophyte formation, subchondral osteosclerosis, gradual articular cartilage damage, and cartilage loss induced by imbalanced exocellular matrix synthesis and katabolism [2, 3]. There are substantial causative factors of arthritis, like joint injury, joint or limb dysplasia, infectious disease, corpulency, age, and gene factors [4, 5]. Admittedly, OA can be a silent illness for a long time before the emergence of representative symptoms and radiography variations, and in such long-term subclinical phase, impairment to articular cartilages might have happened and become nonreversible [6, 7]. Hence, developing new diagnosis biomarkers reflecting the damage of articular cartilages is appealing for the timely diagnoses and therapies of OA.

The recent high-flux genetic microarray analysis of specimens from sufferers and normal individuals enables us to investigate various diseases at diverse levels from somatic mutations and copy number variations to genomic expressions at the transcriptomic level, along with epigene variations [8, 9]. Recently, many specific genes have been discovered to participate in the progression of OA. For instance, it was reported that the expression level of receptor-interacting protein 3(RIP3) was considerably higher in impaired cartilages from OA sufferers in contrast to normal cartilages. In the murine model, the overexpression of adenoviral RIP3 hastened cartilage impairment, but depleted Rip3 decreased DMM-triggered OA etiopathogenesis. TRIM24-RIP3 axis disturbance facilitated chronic OA via stimulating RIP3 kinase, which reveals that the treatment manipulation of such pathway can offer novel enlightenment for the treatment of OA [10]. Shi et al. revealed that the expression of UHRF1 (ubiquitin like with plant homeodomain and ring finger domains 1) was elevated in human OA cartilages, in contrast to healthy cartilages. The knockout of UHRF1 reinforced cellular autophagic activity to defend cartilage cells against programmed cell death in OA via the PI3K/AKT/mTOR signal path [11]. These finding suggested the important roles of some function genes in OA progression. However, the diagnostic value of many genes has not been investigated in OA.

In this study, we aimed to identify novel diagnostic genes for OA based on bioinformatics and machine learning. We analyzed two GEO datasets (GSE55235 and GSE55457) to determine DEGs between OA and healthy specimens. Then, we analyzed their diagnostic value in OA based on machine learning. Finally, we confirmed our findings based on GEO datasets using our cohort via RT-PCR. Our findings provided novel critical genes involved in the progression of OA.

## 2. Materials and Methods

### 2.1. Clinical Specimens

Blood samples were acquired from spinal OA patients (*n* = ten, 3 females and 7 males, aged between 57 and 70 years). Healthy blood samples were obtained from sufferers receiving the amputation with no OA or rheumatoid arthritis (RA) (*n* = ten, 4 females and 6 males, aged between 33 and 52 years). The entire cartilage samples were collected in accordance with the diagnosis standards of spinal OA of the Orthopaedic Society of the Chinese Medical Association. Every sufferer offered written informed consent for the utilization of their tissular specimens. The present research was approved by the Ethical Board of the Second Clinical College of Guangzhou University of Chinese Medicine (LCBL: 10037).

### 2.2. Quantitative Real-Time PCR (qRT-PCR) Assay

Overall RNA was abstracted from OA samples and normal samples via the TRIZOL reagent (Invitrogen) as per the supplier's specification. For qRT-PCR, RNA was converted into cDNA via reverse transcription from 1 *μ*g overall RNA which was subjected to reverse transcription in an eventual volume of 20 *μ*l via stochastic primers and a Reverse Transcriptional Tool (Takara, PRC). As per the supplier's specification, the reverse transcriptional process was completed at 37°C for 15 min and afterwards at 85°C for 5 s. qRT-PCR analysis was completed via a normal protocol from Power SYBR Green (Takara, PRC). Every protocol was completed as per the supplier's specification. The Δct results were normalised to the values of glyceraldehyde-3-phosphate dehydrogenase (GAPDH). qRT-PCR analysis and data acquisition were completed via an ABI 7500 apparatus. All specimens were studied for three times. The primers were as follows: APOLD1: forward (5′-AGAGATGTAACCCAACTCGTTCA-3′) and reverse (5′-CAGGGGAAGGTGCATCCTC-3′); EPYC: forward (5′-AGGAGGAGGAATCTACTCCCA-3′) and reverse (5′-CAGCGGAGGAATAGCATCAAG-3′); and GAPDH: forward (5′-GCAAATTCCATGGCACCGT-3′) and reverse (5′-TCGCCCCACTTGATTTTGG-3′).

### 2.3. Microarray Data

To establish the diagnosis model of OA, the mRNA expression profiling data of GSE55235 and GSE55457 were obtained from GEO (https://www.ncbi.nlm.nih.gov/geo/). GSE55235 contained blood specimens of 10 OA sufferers and 10 normal controls. GSE55457 contained blood specimens of 10 OA sufferers and 10 normal controls. GSE55235 and GSE55457 were on the foundation of the GPL96 [HG-U133A] Affymetrix Human Genome U133A Array. Those 2 datasets were merged into a metadata cohort for integrated analyses as they had the identical platform and are vital for the combination of data from diverse datasets. In addition, the combat function of the “SVA” package of the R program was utilized to realize the removal of batch effects.

### 2.4. DEG Determination and Integrated Microarray Dataset Analyses

The two datasets were merged into a metadata cohort, and the combat functions were utilized to preprocess and eliminate the batch effect. DEGs between OA and healthy samples were determined via the Limma package in R. ∣Log2FC | >1, *p* < 0.05, and false discovery rate (FDR) < 0.05 were thresholds for DEGs. FDR measures the proportion of false discoveries among a set of hypothesis tests called significant.

### 2.5. Functional Enrichment Analyses

Gene Ontology (GO) and the Kyoto Encyclopedia of Genes and Genomes (KEGG) pathway analyses were completed for sufferers between the risk_high_ group and risk_low_ group via the “clusterProfiler” R package [12]. GO terms and KEGG pathways with *p* < 0.05 had significance on statistics. Disease ontology (DO) enrichment analysis was completed on DEGs via the “clusterProfiler” package and DOSE package in R.

### 2.6. Candidate Diagnosis Marker Selection

To the possible diagnostic factors, two machine learning algorithms were applied for the prediction of OA status. The LASSO was a regressive analytical arithmetic utilizing regularisation to ameliorate the forecast accurateness. The LASSO regressive arithmetic was completed via the “glmnet” package in R to determine the genes remarkably related to the discriminative power of OA and healthy specimens. Support vector machine (SVM) is a monitored machine learning technology extensively used for categorization and regressive analysis. For the purpose of avoiding overfit, an RFE arithmetic was utilized to screen the optimum genes from the metadata cohort. Hence, for the sake of identifying the gene set with the greatest discrimination ability, SVM recursive feature elimination (SVM-RFE) was utilized to screen suitable characteristics.

### 2.7. CIBERSORT Analysis

The computation approach of CIBERSORT (http://cibersort.stanford.edu/) is a deconvolutional arithmetic on the foundation of genetic expressions, and it is utilized to assess the variations of a gene group with respect to the rest of genes within a specimen. By virtue of the CIBERSORT arithmetic, our team identified the immunoresponses of 22 immune cells and assessed the association between those immune cells and the expression of critical genes in normal samples and OA samples. The primary objective of the present research was to identify the association between those immune cells.

### 2.8. Statistical Analysis

Student's *t*-tests were utilized to contrast the genetic expressions between OA specimens and neighboring healthy specimens. For the sake of examining the categorization effects of critical genes on OA and healthy specimens, ROC curves and AUC were computed via the R package “pROC.” Statistical analysis was acquired via the R program 3.5.3 and Prism (GraphPad Prism, USA). A significant difference was considered statistically when ^∗^*p* < 0.05, ^∗∗^*p* < 0.01, ^∗∗∗^*p* < 0.001, or ^∗∗∗∗^*p* < 0.0001.

## 3. Results

### 3.1. Determination of DEGs in OA

**Figure 1:**
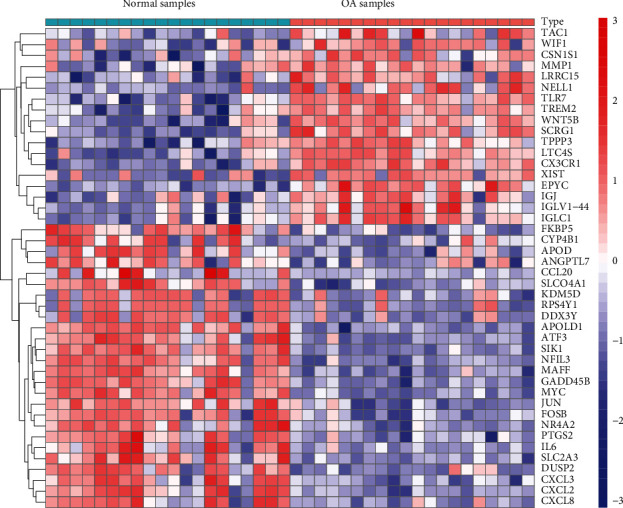
DEGs between OA and healthy specimens.

Data from an overall 20 OA and 20 controls from 2 GEO datasets (GSE55235 and GSE55457) were studied in a retrospective manner in the present research. The DEGs of the metadata were studied via the Limma package after the removal of batch effect. An overall 44 DEGs were identified: 18 genes were remarkably regulated upward and 26 genes were distinctly regulated downward (Figure 1(a)).

### 3.2. Functional Enrichment Analyses

**Figure 2:**
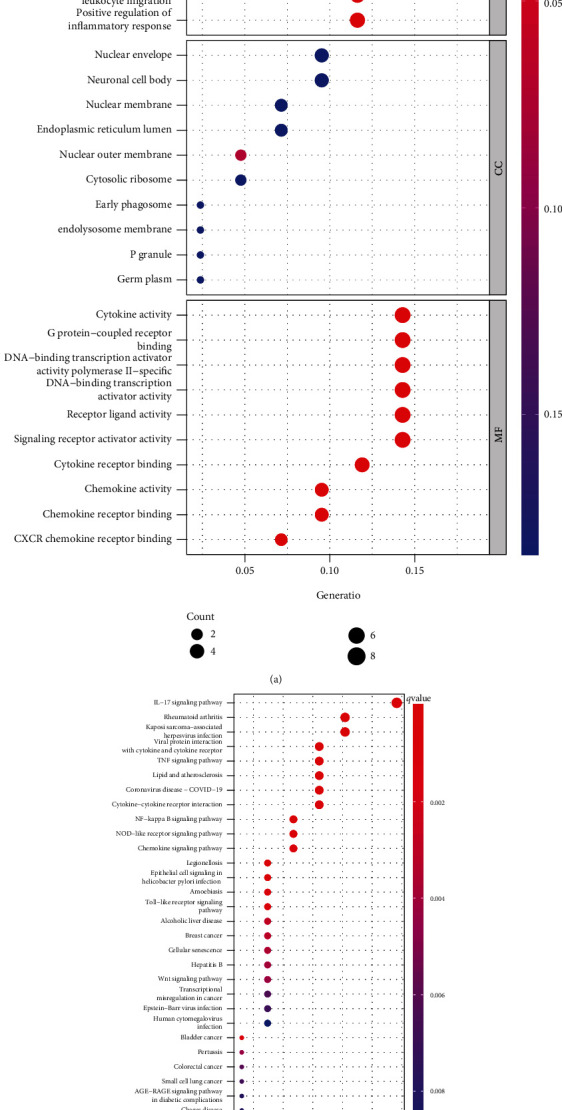
GO analysis (a) and KEGG analysis (b) of 44 DEGs via the ClusterProfile.

For the sake of investigating the biofunction of 44 DEGs in OA, the 44 genes were selected to complete GO analysis and KEGG analysis via the ClusterProfile R package. The outcomes revealed that 44 DEGs were mainly involved in reaction to LPS, reaction to bacteria-originated molecules, myeloid white blood cell migration, cell reaction to chemokine, regulation of white blood cell migration, cell reaction to molecule of bacterial origin, nuclear envelope, neuronal cell body, nuclear membrane, cell factor activity, G protein-coupled acceptor binding, and acceptor ligand activities (Figure 2(a)). Meanwhile, KEGG assays revealed that pathways were significantly enriched including IL-17 signal path, rheumatoid arthritis, TNF signal path, and lipid and atherosclerosis (Figure 2(b)).

### 3.3. Determination and Verification of Diagnosis Markers

**Figure 3:**
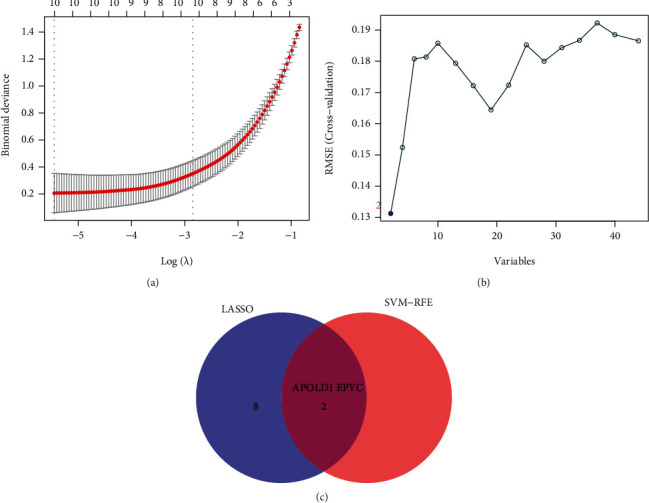
Selection of diagnosis marker candidates for OA: (a) tuning feature screening in the LASSO model; (b) a plot of biological marker screening via the SVM-RFE arithmetic; (c) Venn graph displaying 4 diagnosis biomarkers shared by LASSO and SVM-RFE.

Two diverse arithmetics were utilized to select underlying biological markers. The DEGs were identified via the LASSO regressive arithmetic, which caused the determination of 10 variates as diagnosis markers for OA (Figure 3(a)). A subset of 5 characteristics among the DEGs was identified via the SVM-RFE arithmetic (Figure 3(b)). The 2 overlap characteristics (APOLD1 and EPYC) between those 2 arithmetics were eventually chosen (Figure 3(c)). The above two genes may be critical genes involved in OA progression.

### 3.4. The Expression and Diagnosis Significance of APOLD1 and EPYC in OA

**Figure 4:**
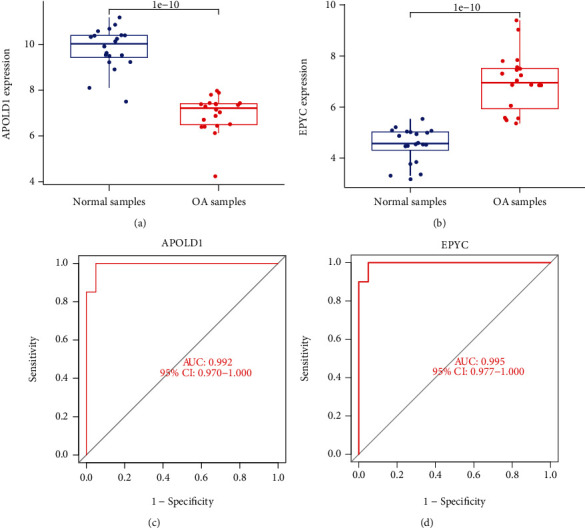
The expression and diagnosis significance of APOLD1 and EPYC in OA: (a) APOLD1 expression was distinctly downregulated in OA samples; (b) EPYC expression was distinctly upregulated in OA samples; (c, d) ROC assays for APOLD1 and EPYC in OA.

Our team discovered that the expression level of APOLD1 was distinctly downregulated in OA samples vs. healthy samples (Figure 4(a)), while EPYC expression was significant upregulated in OA samples (Figure 4(b)). To further explore the diagnostic value of APOLD1 and EPYC, we performed ROC assays. We found that four genes exhibited a strong ability in screening OA samples from normal samples, including APOLD1 (Figure 4(c), AUC = 0.992) and EPYC (Figure 4(d), AUC = 0.995).

### 3.5. APOLD1 and EPYC Are Related to Immunocyte Infiltration Levels

**Figure 5:**
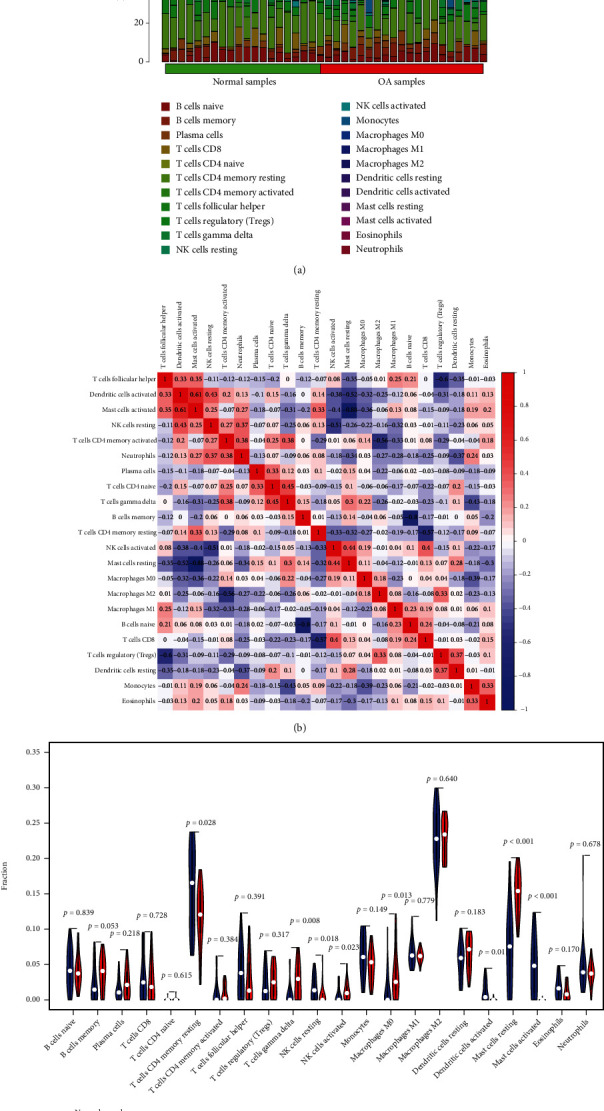
(a, b) The percentage of the 22 immunocytes identified via the CIBERSORT arithmetic. (c) The diversities in the architecture of immunocytes between healthy and OA specimens.

**Figure 6:**
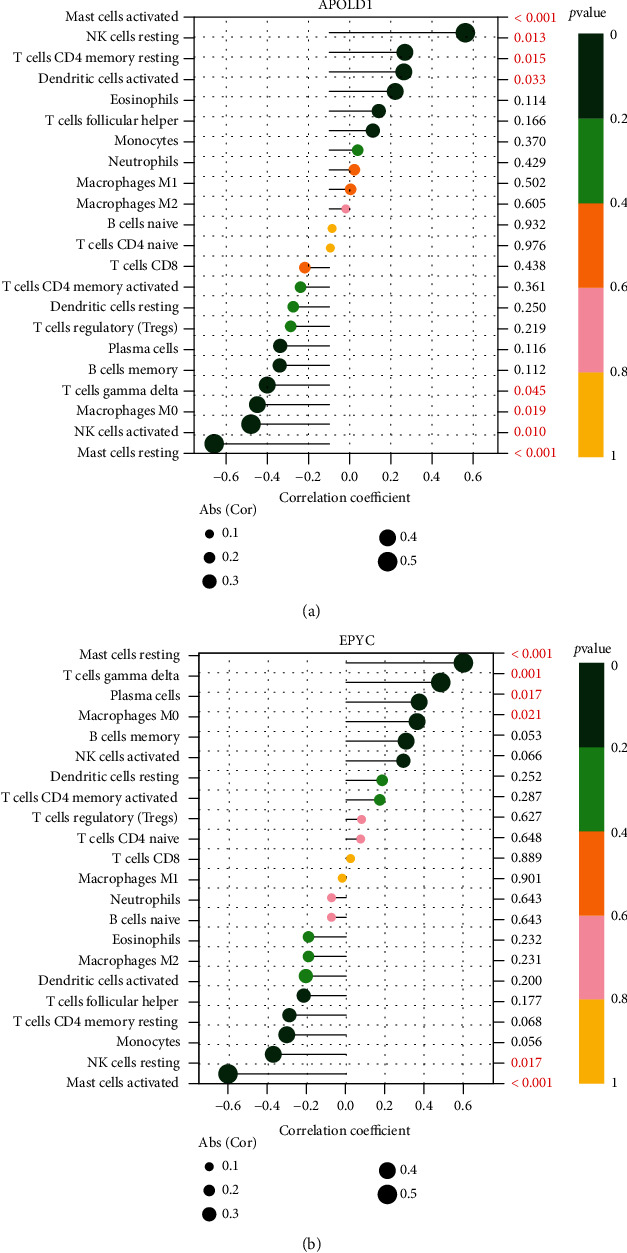
Correlation between APOLD1 (a), EPYC (b), and infiltrating immune cells in OA and normal samples.

Infiltration of associated immunocytes in the TME is an independent prediction factor of OS and prognoses. Hence, our team studied the coefficients of APOLD1 and EPYC and immunocyte infiltration status of OA and normal samples to identify how immunocyte infiltration status is correlated with the expression level of APOLD1 and EPYC. Our team investigated the features of immunocytes via the CIBERSORT approach. Its composition on OA and normal specimens and the relationship among immunocytes are displayed in Figures 5(a) and 5(b). In addition, we observed that the levels of T cells gamma delta, T cells CD4 memory resting, NK cells resting, macrophages M0, dendritic cells (DCs) stimulated, mastocytes resting, and mastocytes stimulated exhibited a dysregulated level between normal samples and OA samples (Figure 5(c)). Moreover, we further explored the relationship between the expressions of APOLD1 and EPYC and immunity infiltrating levels. As shown in Figure 6(a), APOLD1 was correlated with mastocytes stimulated, NK cells resting, T cells CD4 memory resting, DCs stimulated, T cells gamma delta, macrophages M0, NK cells stimulated, and mastocytes resting. Moreover, we found that EPYC was correlated with mastocytes stimulated, NK cells resting, T cells CD4 memory resting, DCs stimulated, T cells gamma delta, macrophages M0, NK cells stimulated, and mastocytes resting (Figure 6(b)). Our findings suggested that APOLD1 and EPYC may be involved in OA progression via regulating several immune cells.

### 3.6. The Identification of the Expression of Four Diagnostic Genes in Our Cohort

**Figure 7:**
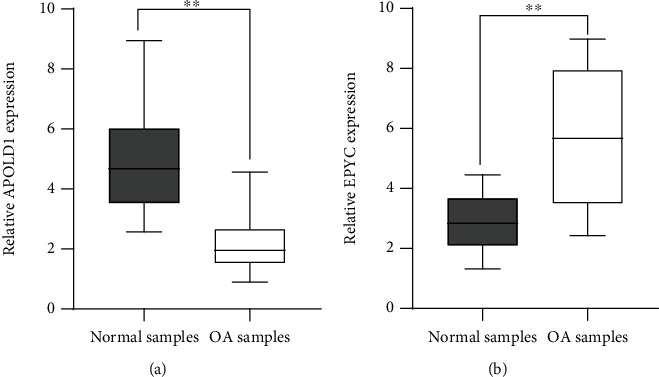
RT-PCR for the levels of APOLD1 (a) and EPYC (b) in OA samples and normal samples from our cohort. The experiments were repeated three times, and each experiment was triplicated. ^∗∗^*p* < 0.01.

Moreover, our team completed RT-PCR to determine the expressions of APOLD1 and EPYC in OA patients and healthy participants. Our team discovered that the expression level of APOLD1 was distinctly decreased in OA samples compared with normal samples (Figure 7(a)). However, the expression level of EPYC was distinctly upregulated in OA samples vs. healthy samples (Figure 7(b)). Thus, our findings suggested that APOLD1 and EPYC may be used as critical diagnostic biomarkers for OA.

## 4. Discussion

OA is the most commonly seen type of arthritis, influencing substantial individuals across the globe [13]. Corpulency elevates the burdens on joints like knees, which elevates stress and can accelerate the impairment of cartilages [14]. Timely diagnoses of OA are vital for the management of such illness, whereas such an early diagnostic tool is still lacking in clinical practice [15, 16]. Herein, we analyzed GEO datasets and identified 44 DEGs between OA specimens and healthy specimens. The results of GO assays indicated that 44 DEGs were mainly involved in reaction to LPS, reaction to bacteria-originated molecules, myeloid white blood cell migration, cell reaction to chemokine, regulation of white blood cell migration, cell reaction to molecule of bacterial origin, nuclear envelope, neuronal cell body, nuclear membrane, cell factor activity, G protein-coupled acceptor binding, and acceptor ligand activity. KEGG pathway analyses revealed that pathways were remarkably sponged, which involved IL-17 signal path, RA, TNF signal path, and lipid and atherosclerosis. These findings suggested that they are positively involved in the inflammatory process. These genes might be vital for the development of OA.

To screen potential diagnostic biomarkers for OA, we performed two machine learning algorithms by the use of the above 44 DEGs, and only two genes (APOLD1 and EPYC) were identified. APOLD1 (Apolipoprotein L Domain-Containing 1) is an endotheliocyte early response protein which might be vital for the modulation of endotheliocyte signal paths and blood vessel functions. To date, the function of APOLD1 in disease progressions was rarely reported. EPYC is a proteoglycan and one of the type III SLRPs. Its gene harbors 7 exons, and exons 3 and 7 harbor underlying mucopolysaccharide attachment spots. The expression of EPYC was discovered in cartilages, ligaments, placentas, and other tissular samples and was vital for the developmental process of cartilages and was pivotal for sustaining joint completeness [17, 18]. Insufficient expressing level of EPYC can facilitate hearing damage and corneal dystrophy [19, 20]. Nevertheless, the expression and role of EPYC in OA are still elusive. Herein, our team firstly reported that EPYC and APOLD1 exhibited a dysregulated level between OA samples and normal samples. Moreover, ROC assays confirmed their strong abilities in screening OA samples from normal samples. Importantly, in our cohort, our team verified that the expressing level of APOLD1 was distinctly downregulated in OA samples vs. healthy samples, while EPYC expression was significantly upregulated in OA samples. Our findings suggested APOLD1 and EPYC as potential diagnostic biomarkers for OA.

Recently, mounting researches have revealed that immunocyte infiltration was vital for the onset and developmental process of OA [21, 22]. OA joints have been discovered to display an evident feature of CD4+ T cell infiltration. CD4+ T cells facilitated the polarisation of stimulated Th1 cells and elevated the excretion of immune regulatory cell factors [23, 24]. Such local inflammatory event aggravated the OA process. Rosshirt and his group revealed that OA joints presented with immunocyte infiltration, like CD16+CD56+ natural killer cells, CD8+ T cells, CD4+ T cells, and CD14+ macrophages [25]. Hence, from the angle of the immunosystem, evaluating the infiltration of immunocytes and identifying the diversities in the constituents of infiltrating immunocytes were imperative for revealing the molecule-level causal link beneath OA and designing novel immune therapy targets. In this study, we found that APOLD1 was correlated with mastocytes stimulated, NK cells resting, T cells CD4 memory resting, DCs stimulated, T cells gamma delta, macrophages M0, NK cells stimulated, and mastocytes resting. Moreover, we found that EPYC was correlated with mastocytes stimulated, NK cells resting, T cells CD4 memory resting, DCs stimulated, T cells gamma delta, macrophages M0, NK cells stimulated, and mastocytes resting. Therefore, our team estimated that APOLD1 and EPYC were associated with the occurrence and progress of OA via regulating several immune cells. Those hypotheses needed more researches to reveal the intricate interplay between genes and immunocytes.

There are still some limitations to be acknowledged. First, the specimen size is comparatively small; large-scale clinical trial tests are required. Second, data herein can merely support the correlative analyses between OA and immunocytes, rather than revealing the causality.

## 5. Conclusion

Bioinformatics and experimental data suggested that APOLD1 and EPYC are key DEGs in OA in contrast to healthy specimens. The findings in the present research offered enlightenment for revealing the potential molecule-level causal links of synovial lesion and provided an underlying target for the immunotherapy of OA.

## Data Availability

The data used to support the findings of this study are available from the corresponding author upon request.
